# Prevalence of Cervical Cancer among Cervical Biopsies in a Tertiary Care Center

**DOI:** 10.31729/jnma.5060

**Published:** 2020-07-31

**Authors:** Ganesh Parajuli, Pravakar Dawadi, Sabina Khadka

**Affiliations:** 1 Department of Pathology, Nepalese Army Institute of Health Sciences, Sanobharyang, Kathmandu, Nepal; 2 Nepalese Army Institute of Health Sciences, Sanobharyang, Kathmandu, Nepal

**Keywords:** *cervical cancer*, *mortality*, *prevalence*, *screening*

## Abstract

**Introduction:**

Cervical cancer is one of the most common cancer among the female population in Nepal. The incidence and mortality rate due to cervical cancer is higher in developing countries like Nepal due to a lack of proper screening and early diagnosis. This study aims to find out the prevalence of cervical cancer among cervical biopsies in a tertiary care center.

**Methods:**

A descriptive cross-sectional study was conducted among the hospital records of cervical biopsies from the department of pathology of Shree Birendra Hospital from 1st May 2018 to 30th April 2019. Ethical approval was taken from the Institutional Review Committee in February 2020. This study was conducted among 146 cervical biopsies by using convenience sampling method. Point estimate at 95% Confidence Interval was calculated along with frequency and proportion for binary data. Data were analyzed using excel 2016 software.

**Results:**

The prevalence of cervical cancer among 146 cases included in our study is found to be 6 (4.11%) at 95% Confidence Interval (0.90-7.32). Among those cases of cervical cancer, 4 (66.67%) were squamous cell carcinoma, 1 (16.67%) was adenocarcinoma, and 1 (16.67%) was of other type. Maximum cases of cervical cancer were prevalent among higher age groups.

**Conclusions:**

Cervical cancer-related morbidity and mortality are different in different parts of the world. It's burden is primarily seen in developing countries where there is a lack of effective screening programs.

## INTRODUCTION

Cervical cancer is one of the most common cancer among females in Nepal. It is more prevalent in sexually active females, where Human papilloma virus (HPV) is recognized as the prime causative agent.^1^ It is the second most common cancer type in females in South East Asia region and a major cause of cancer-related mortality among females of low and middle-income countries like Nepal.^2^

There is no population-based cancer registry program to access the prevalence, morbidity, and mortality of any cancer, including cervical cancer. A number of hospital-based cancer registries contribute to the data.^3^ Cervical cancer screening programs can contribute to reducing its incidence and mortality.^4^ The information regarding the prevalence of cervical cancer will help formulate an action plan for effective screening and management.

The aim of this study is to find out the prevalence of cervical cancer among cervical biopsies in a tertiary care center.

## METHODS

A descriptive cross-sectional study was conducted among the hospital records of cervical biopsies from the Department of Pathology of Shree Birendra Hospital from 1st May 2018 to 30th April 2019. Ethical approval was taken from the Institutional Review Committee in February 2020.

The sample size was calculated using the following formula,

$$\begin{array}{ll} \text{n} & \text{= }{\text{Z}}^{\text{2}}\times \text{p}\times \text{(1}-\text{p)}/{\text{e}}^{\text{2}}\\ & \text{= }{\left(1.96\right)}^{\text{2}}\times (0.05)\times (1-0.05)/{(\text{0.05)}}^{\text{2}}\\ & \text{= }72.99\\ & =73 \end{array}$$

where,

n = required sample size,
Z = 1.96 at 95% Confidence Interval
p = prevalence, 5% (educated guess)
q = 1-p
e = margin of error, 5%

Since we used the convenience sampling method, we have doubled our sample size, i.e. 146, to avoid the bias associated with the sampling. The cervical biopsies received at the department of pathology of Shree Birendra Hospital with the definite diagnoses were included in our study. However, those biopsies which could not show any particular diagnosis were excluded. The data were taken from hospital records. The data were analyzed by using excel 2016.

## RESULTS

**Figure 1.**
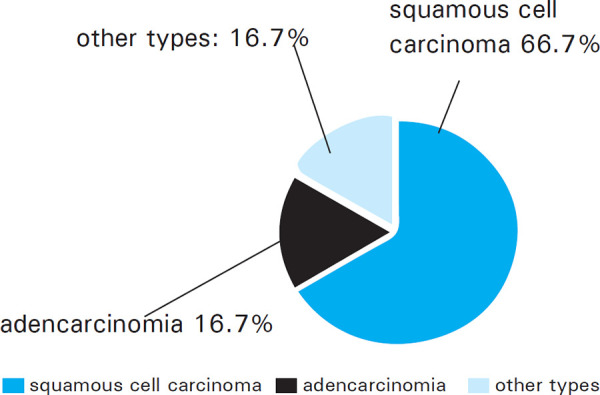
Types of cervical cancer.

Out of 146 cases included in our study, the prevalence of cervical cancer was 6 (4.11%) at a 95% Confidence Interval (0.90-7.32). Among the cases of cervical cancer, squamous cell carcinoma, adenocarcinoma, and other types of cervical cancer were found to be 4 (66.67%), 1 (16.67%) and 1 (16.67%) respectively (Figure 1).

**Figure 2.**
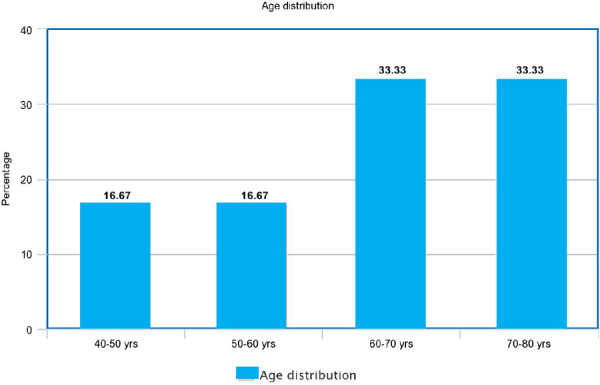
Age distribution of cervical cancer.

The age distribution of cervical cancer patients showed a high prevalence among age groups of 60-80 years of the female population. The cases of cervical cancer among (40-50) years, (50-60) years, (60-70) years and (70-80) years were 1 (16.67%), 1 (16.67%), 2 (33.33%) and 2 (33.33%) respectively (Figure 2).

## DISCUSSION

Cervical cancer is one of the most common cancer in the female population, and its rate varies in different parts of the world. Being the second most common type of cancer among females in the South East Asia region, it accounts for a greater number of cancer-related deaths in countries of this region including Nepal.^2^ The prevalence of 4.11% of cervical cancer in our study also suggests increased risk of cancer-related morbidity and mortality in our setting.

Due to the unavailability of the population-based cancer registry in Nepal, several hospital-based cancer registries serve to provide the relevant data, which is similar in the case of cervical cancer.^3^ Another study also suggests the limited availability of statistical data on cervical cancer in Central Asia.^4^ The limited available data demonstrate a higher prevalence of cervical cancer in a hospital setting. In this scenario, the population-based registry could provide a clear picture of the burden of cervical cancer in the general population.

A study by Shanta V, et al. indicates that there is a higher prevalence of cervical cancer in developing countries^5^ especially in Sub-Saharan Africa and Southern Asia^6^ which is similar to the finding of our study. Another study by Tristen C and Bergstrom S mention that cervical cancer is the leading malignancy in Northeast Brazil.^7^ It is either the first or second most common cancer among females in the developing countries, whereas it is uncommon in developed nations. Lack of proper education and effective screening of high-risk group and poor socio-economic condition in developing countries have led to this high prevalence.^5^

In our study, the ratio of squamous cell carcinoma to other types of cervical cancer is 2:1, which shows a higher proportion of the squamous cell carcinoma among different types of cervical carcinoma. The study by Chan CK, et al. also indicates that the majority of the cervical cancer are of squamous cell carcinoma type.^4^

The findings from our study, along with other studies, suggest that cervical cancer is more common among females of older age groups. The survey by Nkfusai NC, et al. in the Bamenda regional hospital, Northwest region of Cameroon, showed the major prevalence of cervical cancer among the age group of 50-54 years female^8^ whereas in our study it more commonly seen in age groups of 60-80 years female.

The increased rate of cervical cancer is mainly due to ineffective cervical cancer screening.^9^ The developed nations like the United Kingdom, Australia, Canada, Finland, Netherlands, and Singapore have adopted organized programs for cervical cancer screening. However, in most of the Central Asian countries, the Caucasus region, the Russian Federation, and western countries like the former Soviet Union, screening of cervical cancer is opportunistic with low and unreported coverage.^4^ Thus, an effective strategy for screening to cover the majority of the population of the country will help lower the prevalence of cervical cancer.^10^

The findings of our study cannot be generalized to the whole population of Nepal, as this study was conducted in a single hospital in the capital city of Nepal.

## CONCLUSIONS

Cervical cancer-related morbidity and mortality are different in different parts of the world. Its burden is primarily seen in developing countries where there is a lack of effective screening programs. Thus, the planning and implementation of effective screening programs and the enhancement of public awareness regarding cancer will substantially decrease the prevalence of cervical cancer in the general population.

## Conflict of Interest

**None.**
